# Diurnal, seasonal, and sex patterns of heart rate in grip-restrained African giant rats (*Cricetomys gambianus*, Waterhouse)

**DOI:** 10.14814/phy2.12581

**Published:** 2015-10-14

**Authors:** Tavershima Dzenda, Joseph O Ayo, Victor O Sinkalu, Lukuman S Yaqub

**Affiliations:** Department of Veterinary Physiology, Ahmadu Bello UniversityZaria, Nigeria

**Keywords:** Diurnal rhythm, giant rats, heart rate, Savannah, season, sex

## Abstract

This study was carried out to determine heart rate (HR) values, including diurnal, seasonal, and sex patterns, in the African giant rat (*Cricetomys gambianus*, Waterhouse). HR was measured using stethoscope in grip-restrained African giant rats of either sex (103 bucks and 98 does), live-trapped from a tropical Savannah, and caged individually in the laboratory during the harmattan (cold-dry), hot-dry, and rainy seasons over a 3-year period. The HR fluctuated between 90 and 210 beats per minute (bpm) throughout the study period. Diurnal changes in HR (mean ± SEM) during the hot-dry and rainy seasons were nonsignificant (*P* > 0.05), but the morning and afternoon values differed (*P *<* *0.01) during the cold-dry season. The HR varied (*P *<* *0.05) among seasons, with peak, nadir, and moderate values recorded during the cold-dry (165.8 ± 0.51 bpm), hot-dry (153.1 ± 0.74 bpm), and rainy (163.4 ± 0.70 bpm) seasons, respectively. Mean HR of bucks was lower than that of does during the cold-dry (*P *<* *0.0001) and hot-dry (*P *<* *0.01) seasons, but sex difference during the rainy season was insignificant (*P *>* *0.05). Overall, mean HR was lower (*P *<* *0.0001) in bucks (158.8 ± 0.53 bpm) than in does (164.8 ± 0.53 bpm). In conclusion, values of HR in African giant rats are shown for the first time. Season, sex, and daytime influenced the HR, and should be considered during clinical evaluations of the rats.

## Introduction

The relationship between the animal and its environment is best studied based on the knowledge of the physiological constants, maintained by self-regulating processes of the animal, such as body temperature, respiratory rate, and heart rate (HR) (Ayo et al. 2014). The HR, which is an indicator of cardiovascular activity, is defined as the rate of heart beat, expressed in beats per minute (bpm). Although the paraventricular nucleus in the hypothalamus is an established center of cardiovascular control in rats (Feetham and Barrett-Jolley 2014), the HR may be altered rapidly due to inputs from environmental cues (zeitgebers), such as ambient temperature and photoperiod, psychosocial factors like housing, or due to intense activity, including feeding, by the animal (van den Buuse 1999; Swoap et al. 2004; Azar et al. 2011; Carnevali and Sgoifo 2014). A modest diurnal rhythm of resting HR was described in some rodents, including rats (Sei et al. 1997) and mice (Sei et al. 2008), which was independent of locomotor activity (Sheward et al. 2010); and the rhythm of activity itself was modulated mainly by photoperiod and ambient temperature, and to a lesser degree by food availability (Refinetti 2015). The ambient temperature was, perhaps, a weaker zeitgeber than photoperiod (Refinetti 2010), but in the tropics the former was reported (Kowal and Knabe 1972) to fluctuate more widely than the later. The HR of rats (Chambers et al. 2000; William et al. 2002) and mice (Sun et al. 2002; Swoap et al. 2004) was shown to be inversely related to ambient temperature. Season was also reported to influence the HR in rabbits (Abdelatif and Saeed 2009), and significant correlations between HR and seasonal feed consumption patterns were observed in the reindeer (Mesteig et al. 2000). Stupfel and Costagliola (1979) demonstrated that HR in Sprague Dawley rats during most part of their lives always had higher values in females than males, with significant correlations between HR and live weight.

The African giant rat, AGR (*Cricetomys gambianus*, Waterhouse – 1840), is Africa’s largest muroid rodent species, a popular and highly priced exotic pet in Europe (Cooper 2008, 2014), also used for meat in Africa (Ajayi 1977) and odor detection for humanitarian (Verhagen et al. 2003), diagnostic (Mahoney et al. 2014a), and immigration (Mahoney et al. 2014b) purposes. The influences of daytime, season, and/or sex on the body temperature, live weight, feed and water consumptions, and respiratory rate of the AGR were described previously (Dzenda et al. 2011a,b, 2013, 2015). The present study examined some factors that may affect the HR in AGRs with the aim of providing baseline data, which are lacking in the available literature. The data may be of value in domestication, clinical evaluation, and improvement of knowledge of environmental impact on the physiology and adaptation of the AGR. The null hypothesis that daytime, season, and/or sex do not influence the HR of AGRs was tested.

## Materials and Methods

### Study area

The study was conducted in Samaru – Zaria (11°10′N, 07°38′E), located in the Northern Guinea Savannah zone of Nigeria, at an altitude of 686 m above maximum sea level, with a monthly mean photoperiod ranging between 11.49 h in December and 12.76 h in June (Kowal and Knabe 1972). The zone is characterized by three major seasons: the cold-dry or harmattan (November–February), hot-dry (March–April), and rainy (May–October) seasons, with peaks around late December/early January, late March/early April, and late July/August, respectively (Kowal and Knabe 1972; Ayo et al. 2014; Dzenda et al. 2015).

### Animals

#### Ethics

The trap, capture, handling, and management methods utilized in the study conformed to the guidelines of the American Society of Mammalogists (Gannon and Sikes 2007). The procuration of animals, the husbandry, and the experiments also kowtowed to the “European Convention for the Protection of Vertebrate Animals used for Experimental and other Scientific Purposes” (Council of Europe No 123, Strasbourg 1985). All the experimental protocols described were approved by the Ethics Review Committee for Animal Experimentation of Ahmadu Bello University, Zaria.

#### Management

AGRs were live trapped in the Savannah, and managed as described previously (Dzenda et al. 2011a). The sex of each AGR was determined and the rat kept individually in a marked steel cage (Cooper 2008, 2014). Pregnant does were removed and not included in the study. The AGRs were housed in a well-ventilated animal room and preconditioned for at least 2 weeks before the commencement of the experiment. They were fed dry feed pellets (26.56% crude protein, 12.47% ether extract, 10.31% crude fiber, and 8.78% ash). Fresh tap water was given ad libitum using standard commercial rat drinkers.

#### Distribution

A fresh set of AGRs was live trapped and utilized during each season of the 3-year study period. A total of 90 (45 bucks, 45 does), 54 (29 bucks, 25 does), and 57 (29 bucks, 28 does) AGRs were captured and studied during the harmattan, hot-dry, and rainy seasons, respectively. Overall, AGRs of both sexes (103 bucks and 98 does), weighing between 0.8 and 2.0 kg, were considered adult (Ajayi 1977) and used for the study. The number of individual AGRs analyzed per year was 69, 66, and 66 for the 1st, 2nd, and 3rd year, respectively. The yearly distribution of the AGRs by season and sex is shown in Table1.

**Table 1:** Yearly distribution of trapped African giant rats (*Cricetomys gambianus*, Waterhouse) by sex and season during the study period

	Number of African giant rats (*n*)						
	Year 1		Year 2		Year 3		
Season	Male	Female	Male	Female	Male	Female	Total
Harmattan	15	15	15	15	15	15	90
Hot-dry	10	9	9	8	10	8	54
Rainy	10	10	10	9	9	9	57
Total	35	34	34	32	34	32	201

### Heart rate

Each AGR was restrained using the “under the shoulders grip” method (Machholz et al. 2012). The HR of each restrained AGR was recorded as the number of heart beats per minute (bpm) by auscultation with the aid of a stethoscope (3M™ Littman® Classic II S.E.), placed on the left ventro-lateral side of the thorax (Kodesh et al. 2011; Ayo et al. 2014). The HRs of the AGRs were measured in the morning (7–9 h), afternoon (12–14 h), and evening (16–18 h) for 3 days during each season of the 3-year experimental period. Indicated times are West-Central African time (GMT +1). The three experimental days were spread across 3 weeks, that is, 1 day per week, during each season.

### Statistical analyses

Data were analyzed using GraphPad Prism, version 4.03 for Windows (GraphPad Software, San Diego, CA; www.graphpad.com). The data obtained were expressed as mean ± standard error of the mean (mean ± SEM). Diurnal and seasonal variations of HR in the AGRs were analyzed using repeated-measures and one-way analyses of variance, respectively, followed by Tukey’s multiple comparison post hoc tests. Two-tailed, paired Student’s *t*-test was used to determine sex differences in the HR values. The relationships between HR and live weight of the AGRs, and environmental thermal factors, recorded in the experimental room (Dzenda et al. 2011a), including ambient temperature, relative humidity, and temperature–humidity/heat index were established using two-tailed Pearson’s correlation analysis. Values of *P *<* *0.05 were considered significant.

## Results

Maximum, minimum, and range values of HR in the AGRs are shown in Tables2 and 3. The HR fluctuated between 90 and 210 bpm throughout the study period in the AGR bucks (90–207 bpm) and does (96–210 bpm) during the cold-dry (111–210 bpm), hot-dry (90–192 bpm), and rainy (96–198 bpm) seasons, in the morning (105–198 bpm), afternoon (105–207 bpm), and evening (90–210 bpm). Nadir and peak individual values were recorded during the hot-dry and cold-dry seasons, respectively. The overall maximum value of 210 bpm was acquired from a doe AGR during the cold-dry season (Table2) in the evening (Table3), while the overall minimum value of 90 bpm was obtained from a buck AGR during the hot-dry season (Table2) in the evening (Table3). The general range of HR values was less in the morning (93 bpm) and during the cold-dry season (99 bpm), but highest (120 bpm) in the evening (Table3).

**Table 2:** Seasonal and sex variations in maximum, minimum, and range heart rate values of African giant rats (*Cricetomys gambianus*, Waterhouse)

Season	Heart rate (bpm)								
	Male (*n *=* *103)			Female (*n *=* *98)			Overall (*n *=* *201)		
	Maximum	Minimum	Range	Maximum	Minimum	Range	Maximum	Minimum	Range
Harmattan	207	108	99	210	129	81	210	108	102
Hot-dry	189	90	99	192	108	84	192	90	102
Rainy	195	105	90	198	96	102	198	96	102
Overall	207	90	117	210	96	114	210	90	120
Mean ± SEM	197.0 ± 5.29^a^	101.0 ± 5.57^b^	96.00 ± 3.00	200.0 ± 5.29^c^	111.0 ± 9.64^d^	89.00 ± 6.56	200.0 ± 5.29^e^	98.00 ± 5.29^f^	102.0 ± 0.00

For each sex, means (± SEM) of maximum and minimum values with different superscript letters are significantly different

a,b *P *=* *0.001;

c,d *P *<* *0.01;

e,f *P *<* *0.001).

**Table 3:** Diurnal and seasonal variations in maximum, minimum, and range heart rate values of African giant rats (*Cricetomys gambianus*, Waterhouse)

Time of day	Heart rate (bpm)											
	Harmattan season (*n *=* *90)			Hot-dry season (*n *=* *54)			Rainy season (*n *=* *57)			Overall (*n *=* *201)		
	Max	Min	Range	Max	Min	Range	Max	Min	Range	Max	Min	Range
Morning (07–09 h)	195	114	81	192	105	87	198	114	84	198	105	93
Afternoon (12–14 h)	207	125	82	186	105	81	195	108	87	207	105	102
Evening (16–18 h)	210	111	99	192	90	102	195	96	99	210	90	120
Overall	210	111	99	192	90	102	198	96	102	210	90	120
Mean ± SEM	204.0 ± 4.58^a^	116.7 ± 4.26^b^	87.33 ± 5.84	190.0 ± 2.00^a^	100.0 ± 5.00^b^	90.00 ± 6.25	196.0 ± 1.00^a^	106.0 ± 5.29^b^	90.00 ± 4.58	205.0 ± 3.61^a^	100.0 ± 5.00^b^	105.0 ± 7.94

For each season, means (± SEM) of maximum and minimum values with different superscript letters

(a,b) are significantly different (*P *<* *0.01). Max = maximum; Min = minimum.

**Figure 1:**
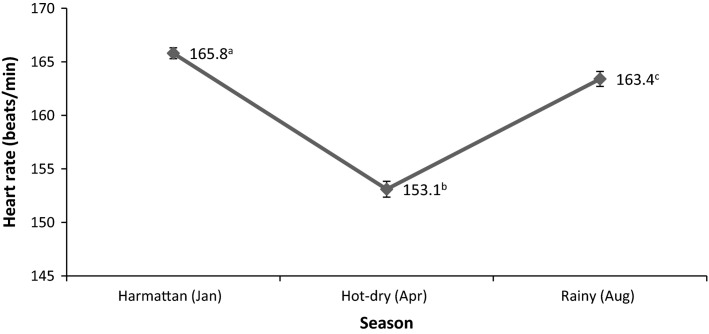
Seasonal variation in heart rate of African giant rats (*Cricetomys gambianus*, Waterhouse). Animal subject numbers (*n*) for each season are reported in Table1. Means (± SEM) with different superscript letters (^a,b,c^) are significantly different (^a,b^ and ^b,c^ *P* < 0.001; ^a,c^ *P* < 0.05).

The mean value of HR obtained from the AGRs differed (*P *<* *0.05) among the three seasons with zenith (165.8 ± 0.51 bpm) and nadir (153.1 ± 0.74 bpm) values recorded during the cold-dry and hot-dry seasons, respectively (*P *<* *0.001); the value for the rainy season (163.4 ± 0.70 bpm) being closer to that of the cold-dry (*P *<* *0.05) than the hot-dry (*P *<* *0.001) seasons (Fig.1).

**Figure 2:**
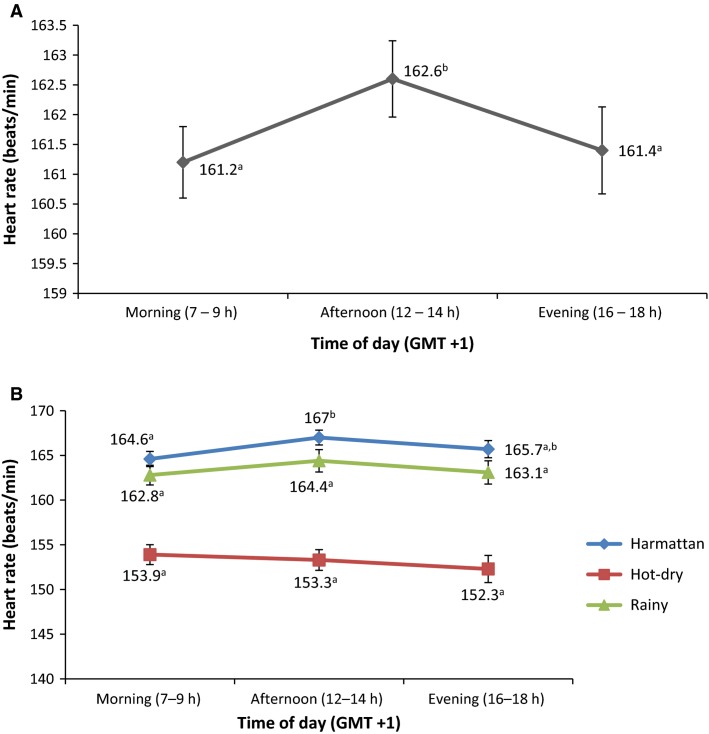
Overall (A) and across season (B) diurnal variations in heart rate of African giant rats (*Cricetomys gambianus*, Waterhouse). Subject numbers (*n*) are reported in Table1. Mean (± SEM) values in the same series with different and/or not sharing superscript letters (^a,b^) are significantly (*P* < 0.05) different.

The overall diurnal mean HR value of the AGRs obtained in the afternoon (162.6 ± 0.64 bpm) was higher (*P *<* *0.05) than in the morning (161.2 ± 0.60 bpm) or evening (161.4 ± 0.73 bpm) (Fig.2A). However, significant diurnal differences were recorded only during the cold-dry season, between the morning (164.6 ± 0.85 bpm) and afternoon (167.0 ± 0.83 bpm) values (Fig.2B).

**Figure 3:**
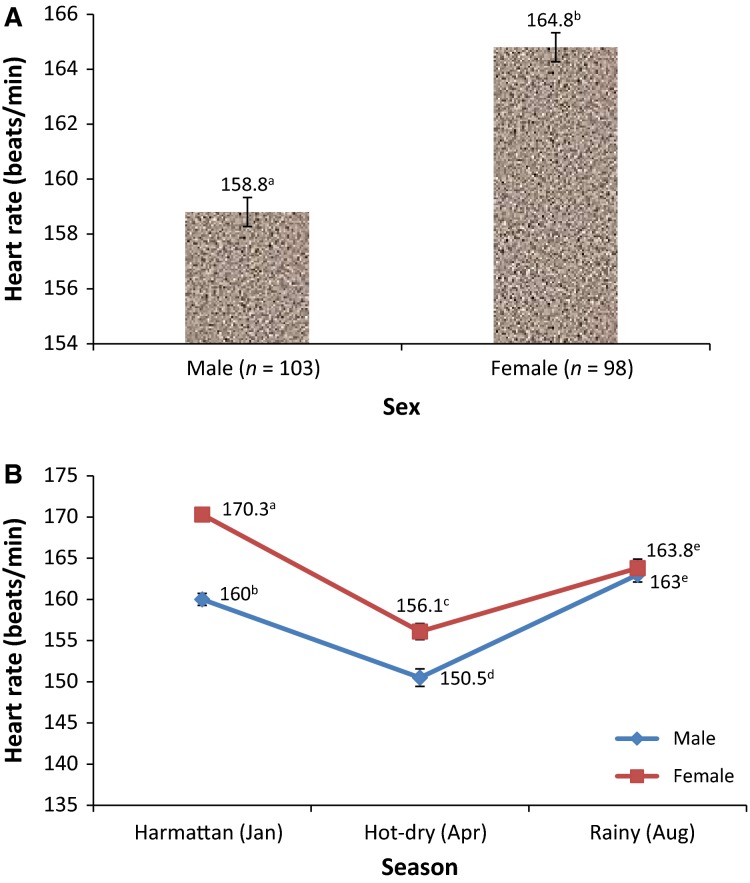
Overall (A) and across season (B) sex variations in heart rate of African giant rats (*Cricetomys gambianus*, Waterhouse). Subject numbers (*n*) for each sex per season are reported in Table1. Mean (± SEM) values with different superscript letters are significantly different (^a,b^ *P* < 0.0001; ^c,d^ *P* < 0.01).

The overall mean HR recorded in the AGRs was significantly (*P *<* *0.0001) lower in AGR bucks (158.8 ± 0.53 bpm), compared to the value obtained in the AGR does (164.8 ± 0.53 bpm) (Fig.3A). The sex difference was, nonetheless, significant during the cold-dry (*P *<* *0.0001) and hot-dry (*P *<* *0.01) seasons, but not during the rainy season (*P *>* *0.05) (Fig.3B).

**Figure 4:**
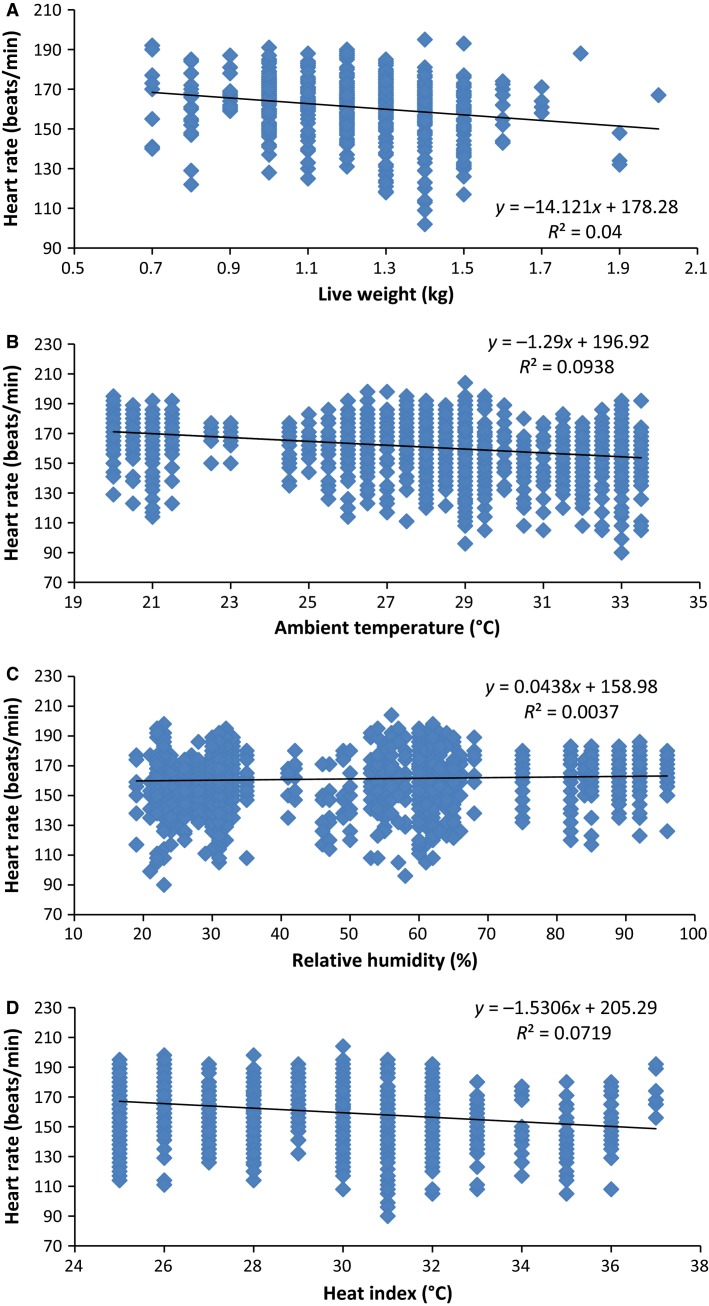
Relationships between heart rate and live weight (A) of the African giant rat (*Cricetomys gambianus*, Waterhouse), and ambient temperature (B), relative humidity (C), and heat index (D) of the experimental room. Each dot in the graphs represents the variable observed in an individual animal subject. For each relationship (A – D) the number of subjects (*n*) = 201, while that of XY pairs = 1,654.

Correlation coefficients (Pearson *r* values) between the HR and live weight in both sexes of the AGRs and the thermal environmental parameters of the experimental room are shown in Table4, while the overall relationships between the variables are illustrated in Figure4. The association between HR and live weight was negative across the sexes (*P *<* *0.0001) and amid doe AGRs (*P *< 0.01), but nonsignificant (*P *>* *0.05) among the bucks. Correlations of the HR with ambient temperature and heat index were also negative and very highly significant (*P *<* *0.0001) in both sexes of the AGRs. The relationship between HR and relative humidity was positive and significant (*P *<* *0.0001) in bucks, but insignificant (*P *>* *0.05) in does of the AGR.

**Table 4:** Correlation coefficients between heart rate and live weight of the African giant rats (*Cricetomys gambianus*, Waterhouse), and environmental thermal parameters of the experimental room

Correlated parameters	Correlation coefficient (Pearson *r*)		
	Male (*n *=* *103; XY pairs = 846)	Female (*n *=* *98; XY pairs = 808)	Overall (*n *=* *201; XY pairs = 1654)
Heart rate and live weight	−0.0692^ns^	−0.1804**	−0.1999****
Heart rate and ambient temperature	−0.2837****	−0.3332****	−0.3063****
Heart rate and relative humidity	0.1720****	−0.0553 ns	0.0610*
Heart rate and heat index	−0.2041****	−0.3356****	−0.2681****

**** *P *<* *0.0001

** *P *<* *0.01

* *P *<* *0.05

ns *P *>* *0.05.

## Discussion

The results showed individual fluctuations in values of the HR over a 3-year period in buck and doe AGRs, during the cold-dry, hot-dry, and rainy seasons, in the morning, afternoon, and evening. The results demonstrated the importance of generating data across seasons and during the hours of the day in both sexes before concluding on baseline/reference values. The minimum–maximum HR values obtained in the AGRs (90–210 bpm) were lower than those reported for laboratory rats (300–500 bpm) (van Zutphen et al. 2001; Azar et al. 2011); apparently due to the larger size of the former (Ajayi 1977; Dzenda et al. 2011b), since HR and live weight are inversely related across species (Detweiler 2010).

A modest diurnal rhythm of HR was observed in grip-restrained AGRs in the present study, which agreed with previous findings in smaller rodents, including laboratory rats (Sei et al. 1997) and mice (Sei et al. 2008; Sheward et al. 2010). The overall diurnal rhythm of the HR observed in the current study was less robust than those reported for the rectal temperature (Dzenda et al. 2011a) and respiratory rate (Dzenda et al. 2015) in the AGR; assenting with the appraisal by Mortola and Lanthier (2004) of the amplitudes of circadian patterns in many mammals, including rats and mice. It was also observed in the present study that the diurnal variation in HR of the AGRs was significant (*P *<* *0.05) only during the cold-dry season, in contrast with the nonsignificant rhythm obtained during the hot-dry and rainy seasons. This finding conceivably reflected the influence of the more robust diurnal ambient temperature pattern prevailing during the cold-dry season (Igono et al. 1982; Dzenda et al. 2011a; Ayo et al. 2014). Likewise, the photoperiodic shift to shorter days during the cold-dry season (Kowal and Knabe 1972) may contribute to the difference in diurnal rhythm of HR observed in the AGR during the season; concurring with the findings of van den Buuse (1999) and Zhang et al. (2000), who reported shifts in circadian rhythm of HR in Sprague Dawley and Wistar rats, respectively, in response to experimental shortening of the light cycle. Thus, ambient temperature and photoperiod may be major environment cues (Cable et al. 2007; Refinetti 2010, 2015) to which the currently observed modest diurnal rhythm of HR in AGRs was entrained.

The HR of the AGRs fluctuated seasonally, with apex and nadir values recorded during the cold-dry and hot-dry seasons, respectively, suggesting that the dry seasons were more stressful to the AGRs compared to the rainy season, during which moderate HR values were obtained. The pattern of seasonal fluctuations in HR was the very opposite of that reported for the ambient temperature (Dzenda et al. 2011a). Hence, the sharp seasonal decline in HR of AGRs during the hot-dry season in the present study may be induced by high ambient temperature (Swoap et al. 2004), resulting in decreased feed and water consumptions (William et al. 2002; Dzenda et al. 2013). Furthermore, the low HR may reflect changes in cardiac output and/or the rate of flow of blood to the gastrointestinal tract, which are influenced by meal size (Barnes et al. 1983; Mesteig et al. 2000). Conversely, the HR rose (*P *<* *0.001) during the rainy season, and was highest in the cold-dry season in consonance with a previously reported (Dzenda et al. 2013) increase in feed and water consumptions in the AGR during the two seasons. This proposition was supported by a very highly significant and negative correlation of the HR in the AGRs with changes in ambient temperature and heat index of the experimental room (Table4). The seasonal pattern of HR observed in the present study was different from that of the rectal temperature (Dzenda et al. 2011a) or respiratory rate (Dzenda et al. 2015), but closely resembled those of feed and water consumptions (Dzenda et al. 2013), suggesting that the later elicited greater cardiovascular response in the AGRs. The finding agreed with that of Mesteig et al. (2000), who observed that the seasonal pattern of HR in reindeer was different from that of body temperature, being influenced by seasonal feeding patterns rather than body temperature. The present finding also agreed with that of Abdelatif and Saeed (2009), who reported higher HR values during winter than summer in the rabbit.

The present results showed that the overall HR of doe AGRs was higher (*P *<* *0.0001) than that of bucks, with a very highly significant and negative relationship between the HR and live weight across the sexes; in accord with the finding of Stupfel and Costagliola (1979), who reported that HR in Sprague Dawley rats during most part of their lives was always higher in females than males, with significant correlations between HR and live weight. Thus, the smaller adult size of doe AGRs (Ajayi 1977; Dzenda et al. 2011b, 2013) may be at least partly responsible for their higher HR values, compared to those of their male counterparts.

It was observed that the sex difference in HR of the AGRs was significant during the cold-dry (*P *<* *0.0001) and hot-dry (*P *<* *0.01) seasons, indicating that either extremes of thermal stress during the dry seasons elicited sex differences in HR adaptive response (Yang et al. 2007), but not (*P *>* *0.05) during the moderately stressful rainy season. The present results agreed with an earlier report (Dzenda et al. 2013) that sex differences in feed and water consumptions of AGRs were significant during the dry seasons, but not during the rainy season; suggesting that feeding and drinking had dominant influences over live weight on the HR of AGRs during the rainy season in the present study. Hence, the HR in the smaller sized doe AGRs was not different from that of their larger sized male counterparts once relative feed and water consumptions approximated between the sexes during the rainy season. The sex variation in HR of AGRs observed in the present study was season dependent, like that of their body temperature (Dzenda et al. 2011a) or respiratory rate (Dzenda et al. 2015), but the seasonal pattern of the sex disparity differed in that the later exhibited the nonsignificant sex difference during the hot-dry, instead of the rainy, season.

Sex difference was also manifested in the relationship between HR and relative humidity, which was significantly positive in AGR bucks but nonsignificant in the AGR does. The results indicate that the AGR bucks adopted cardiovascular adaptive responses (Yang et al. 2007) to the seasonal moisture fluctuations (Ball and Ketterson 2008) prevailing in the tropical Northern Guinea Savannah zone; whereas a previous report (Dzenda et al. 2015) implied that the later, on the other hand, responded to the challenges with respiratory compensations. The sex differences in relationships between physiological indices in the AGR, such as HR and live weight, and thermal environmental parameters are suggestive of sexual dimorphism in thermal adaptability of the AGR (Mogil et al. 2000; Byanet et al. 2015).

A limitation of the current study was the lack of “resting” baseline data from free-moving, unrestrained AGRs; hence, the influence of the grip-restraint method on absolute values of the HR could not be assessed. However, in an appraisal of previous studies, Balcombe et al. (2004) showed that handling and restraint induced significant increases in HR of male and female laboratory rats, indicating that the present baseline values may differ from those that might be obtained from unconstrained AGRs. It was also not feasible with the methodology used in the present study to obtain reasonable values for the time between heart beats in order to deduce HR variability (HRV) in the AGRs. The HRV parameter, which is often used to complement HR as an indicator of autonomic activity (Acharya et al. 2006), was shown in some rodents to be significantly increased/higher (lower sympathetic/higher parasympathetic activity) during summer (Weil et al. 2009), in females (Johnson et al. 2011), during the “inactive period” of the day (Albarwani et al. 2013), and with mild psychological stress (Feetham and Barrett-Jolley 2014). It is conceivable that the HRV in AGRs may be elevated/higher during the rainy season, in does, during the afternoon (since AGRs are nocturnal), and in free-moving, unrestrained rats.

In conclusion, the present study provides baseline data on diurnal, seasonal, and sex variations in the HR of grip-restrained AGRs, which may be useful for clinical evaluation of the rodent. The results suggest the influences of ambient thermal conditions and live weight on the HR patterns in AGRs.

## References

[b1] Abdelatif AM, Saeed IH (2009). Thermoregulation, heart rate and body weight as influenced by thyroid status and season in the domestic rabbit (*Lepus cuniculus*. Middle East J. Sci. Res.

[b2] Acharya UR, Joseph KP, Kannathal N, Lim CM, Suri JS (2006). Heart rate variability: a review. Med. Biol. Eng. Comput.

[b3] Ajayi SS (1977). Live and carcass weights of Giant rat (*Cricetomys gambianus*, Waterhouse) and domestic rabbit (*Oryctolagus cuniculus* L). Afr. J. Ecol.

[b4] Albarwani S, Al-Siyabi S, Tanira MO (2013). Lisinopril indifferently improves heart rate variability during day and night periods in spontaneously hypertensive rats. Physiol. Res.

[b5] Ayo JO, Dzenda T, Olaifa F, Ake SA, Sani I (2014). Diurnal and seasonal fluctuations in rectal temperature, respiration and heart rate of pack donkeys in a tropical Savannah zone. J. Equine Sci.

[b6] Azar T, Sharp J, Lawson D (2011). Heart rates of male and female Sprague-Dawley and spontaneously hypertensive rats housed singly or in groups. J. Am. Assoc. Lab. Anim. Sci.

[b7] Balcombe JP, Barnard ND, Sandusky C (2004). Laboratory routines cause animal stress. J. Am. Assoc. Lab. Anim. Sci.

[b8] Ball GF, Ketterson ED (2008). Sex differences in the response to environmental cues regulating seasonal reproduction in birds. Philos. Trans. R. Soc. Lond. B Biol. Sci.

[b9] Barnes RJ, Comline RS, Dobson A (1983). Changes in the blood flow to the digestive organs of sheep induced by feeding. Q. J. Exp. Physiol.

[b10] van den Buuse M (1999). Circadian rhythms of blood pressure and heart rate in conscious rats: effects of light cycle shift and timed feeding. Physiol. Behav.

[b11] Byanet O, Dzenda T, Obadiah IH (2015). Tail allometry of the grasscutter (*Thryonomys swinderianus*) and African Giant pouched rat (*Cricetomys gambianus*): its functional relevance. World J. Zool.

[b12] Cable NT, Drust B, Gregson WA (2007). The impact of altered climatic conditions and altitude on circadian physiology. Physiol. Behav.

[b13] Carnevali L, Sgoifo A (2014). Vagal modulation of resting heart rate in rats: the role of stress, psychosocial factors, and physical exercise. Front. Physiol.

[b14] Chambers JB, William TD, Nakamura A, Henderson RP, Overton JM, Rashotte ME (2000). Cardiovascular and metabolic responses of hypertensive and normotensive rats to one week of cold exposure. Am. J. Physiol. Regul. Integr. Comp. Physiol.

[b15] Cooper RG (2008). Care, husbandry and diseases of the African Giant rat (*Cricetomys gambianus*. J. S. Afr. Vet. Assoc.

[b16] Cooper RG (2014). The African Giant/Pouched Rat (Cricetomys gambianus) – its physiology, ecology, care & taming.

[b17] Detweiler DK, Macfarlane PW, van Oosterom A, Pahlm O, Kligfield P, Janse M, Camm J (2010). The mammalian electrocardiogram: comprehensive features: interspecies correlations: body size, heart rate and time intervals. Comprehensive electrocardiology, volume 4.

[b18] Dzenda T, Ayo JO, Lakpini CAM, Adelaiye AB (2011a). Diurnal, seasonal and sex variations in rectal temperature of African Giant rats (*Cricetomys gambianus*, Waterhouse). J. Therm. Biol.

[b19] Dzenda T, Ayo JO, Lakpini CAM, Adelaiye AB (2011b). Seasonal and sex variations in live weights of captive African Giant rats (*Cricetomys gambianus*, Waterhouse) in the Northern Guinea Savannah zone of Nigeria. Int. J. Zool. Res.

[b20] Dzenda T, Ayo JO, Lakpini CAM, Adelaiye AB (2013). Seasonal, sex and live weight variations in feed and water consumptions of adult captive African Giant rats (*Cricetomys gambianus*, Waterhouse – 1840) kept individually in cages. J. Anim. Physiol. Anim. Nutr. (Berl).

[b21] Dzenda T, Ayo JO, Lakpini CAM, Adelaiye AB (2015). Diurnal, seasonal and sex influences on respiratory rate of African Giant rats (*Cricetomys gambianus*, Waterhouse) in a tropical Savannah. Wulfenia J.

[b22] Feetham CH, Barrett-Jolley R (2014). NK1-receptor-expressing paraventricular nucleus neurones modulate daily variation in heart rate and stress-induced changes in heart rate variability. Physiol. Rep.

[b23] Gannon WL, Sikes RS (2007). The Animal care and use committee of the American society of mammalogists. Guidelines of the American society of mammalogists for the use of wild mammals in research. J. Mammal.

[b24] Igono MO, Molokwu ECI, Aliu YO (1982). Body temperature responses of Savannah Brown goat to the harmattan and hot-dry seasons. Int. J. Biometeorol.

[b25] Johnson MS, DeMarco VG, Heesch CM, Whaley-Connell AT, Schneider RI, Rehmer NT (2011). Sex differences in baroreflex sensitivity, heart rate variability, and end organ damage in the TGR(mRen2)27 rat. Am. J. Physiol. Heart Circ. Physiol.

[b26] Kodesh E, Zaldivar F, Schwindt C, Tran P, Yu A, Camilon M (2011). A rat model of exercise-induced asthma: a nonspecific response to a specific immunogen. Am. J. Physiol. Regul. Integr. Comp. Physiol.

[b27] Kowal JM, Knabe DT (1972). An agroclimatological atlas of the northern states of Nigeria: with explanatory notes.

[b28] Machholz E, Mulder G, Ruiz C, Corning BF, Pritchett-Corning KR (2012). Manual restraint and common compound administration routes in mice and rats. J. Vis. Exp.

[b29] Mahoney A, Edwards TL, La Londe K, Beyene N, Cox C, Weetjens BJ (2014a). Pouched rats’ (*Cricetomys gambianus*) detection of salmonella in horse faeces. J. Vet. Behav. Clin. Appl. Res.

[b30] Mahoney A, La Londe K, Edwards TL, Cox C, Weetjens BJ, Poling A (2014b). Detection of cigarettes and other tobacco products by Giant African pouched rats (*Cricetomys gambianus*. J. Vet. Behav. Clin. Appl. Res.

[b31] Mesteig K, Tyler NJC, Blix AS (2000). Seasonal changes in heart rate and food intake in reindeer (*Rangifer tarandus tarandus*. Acta Physiol. Scand.

[b32] Mogil JS, Chesler EJ, Wilson SG, Juraska JM, Sternberg WF (2000). Sex differences in thermal nociception and morphine antinociception in rodents depend on genotype. Neurosci. Biobehav. Rev.

[b33] Mortola JP, Lanthier C (2004). Scaling the amplitudes of the circadian pattern of resting oxygen consumption, body temperature and heart rate in mammals. Comp. Biochem. Physiol. Part A.

[b34] Refinetti R (2010). Entrainment of circadian rhythm by ambient temperature cycles in mice. J. Biol. Rhythms.

[b35] Refinetti R (2015). Comparison of light, food, and temperature as environmental synchronizers of the circadian rhythm of activity in mice. J. Physiol. Sci.

[b36] Sei H, Furuno N, Morita Y (1997). Diurnal changes of blood pressure, heart rate and body temperature during sleep in the rat. J. Sleep Res.

[b37] Sei H, Oishi K, Chikahisa S, Kitaoka K, Takeda E, Ishida N (2008). Diurnal amplitudes of arterial pressure and heart rate are dampened in clock mutant mice and adrenalectomized mice. Endocrinology.

[b38] Sheward WJ, Naylor E, Knowles-Barley S, Armstrong JD, Brooker GA, Seckl JR (2010). Circadian control of mouse heart rate and blood pressure by the suprachiasmatic nuclei: behavioural effects are more significant than direct outputs. PLoS One.

[b39] Stupfel M, Costagliola D (1979). Life-long variations in heart rates in SPF Sprague-Dawley rats of both sexes. Pflügers Arch. Eur. J. Physiol.

[b40] Sun Z, Cade R, Morales C (2002). Role of central angiotensin II receptors in cold-induced hypertension. Am. J. Hypertens.

[b41] Swoap SJ, Overton JM, Garber G (2004). Effect of ambient temperature on cardiovascular parameters in rats and mice: a comparative approach. Am. J. Physiol. Regul. Integr. Comp. Physiol.

[b42] Verhagen R, Cox C, Machangu R, Weetjens B, McLean IG, Billet M (2003). Preliminary results on the use of *Cricetomys* rats as indicators of buried explosives in field conditions. Mine detection dogs: training, operations and odour detection.

[b43] Weil ZM, Norman GJ, DeVries AC, Berntson GG, Nelson RJ (2009). Photoperiod alters autonomic regulation of the heart. Proc. Natl. Acad. Sci. USA.

[b44] William TD, Chambers JB, Henderso RP, Rashotte ME, Overton JM (2002). Cardiovascular responses to caloric restriction and thermoneutrality in (57BL16J) mice. Am. J. Physiol. Regul. Integr. Comp. Physiol.

[b45] Yang JN, Tiselius C, Daré E, Johansson B, Valen G, Fredholm BB (2007). Sex differences in mouse heart rate and body temperature and in their regulation by adenosine A1 receptors. Acta Physiol. (Oxf).

[b46] Zhang B-L, Zannou E, Sannajust F (2000). Effects of photoperiod reduction on rat circadian rhythms of BP, heart rate, and locomotor activity. Am. J. Physiol. Regul. Integr. Comp. Physiol.

[b47] van Zutphen LF, Baumans V, Beynen AC (2001). Principles of laboratory animal science: a contribution to the humane use and care of animals and to the quality of experimental results.

